# Klein’s Plastic Dentine

**Published:** 1899-11

**Authors:** 


### KLEIN'S PLASTIC DENTINE.

To the Dental Profession:

Dr. E. A. Bogue, of New York and Paris, has informed us that his name has been used in support of this preparation without his knowledge or authority.

We greatly regret that this error has been unwittingly circulated by us, and have withdrawn Dr. Bogue's name from the pamphlet.

Claudius Ash & Sons (Limited).

